# Structural and biochemical characterization of the prenylated flavin mononucleotide-dependent indole-3-carboxylic acid decarboxylase

**DOI:** 10.1016/j.jbc.2022.101771

**Published:** 2022-02-24

**Authors:** Deepankar Gahloth, Karl Fisher, Karl A.P. Payne, Matthew Cliff, Colin Levy, David Leys

**Affiliations:** Manchester Institute of Biotechnology, University of Manchester, Manchester, UK

**Keywords:** decarboxylase, prFMN, carboxylation, flavin, crystal structure, enzyme, protein purification, molecular biology, AnInD, indole-3-carboxylate decarboxylase from *Arthrobacter nicotianae*, CAR, carboxylic acid reductase, I3C, indole-3-carboxylic acid, Ni-NTA, Ni-nitriloacetic acid, prFMN, prenylated flavin mononucleotide, SAXS, small-angle X-ray scattering, VdcCD, vanillic acid decarboxylase

## Abstract

The ubiquitous UbiD family of reversible decarboxylases is implicated in a wide range of microbial processes and depends on the prenylated flavin mononucleotide cofactor for catalysis. However, only a handful of UbiD family members have been characterized in detail, and comparison between these has suggested considerable variability in enzyme dynamics and mechanism linked to substrate specificity. In this study, we provide structural and biochemical insights into the indole-3-carboxylic acid decarboxylase, representing an UbiD enzyme activity distinct from those previously studied. Structural insights from crystal structure determination combined with small-angle X-ray scattering measurements reveal that the enzyme likely undergoes an open-closed transition as a consequence of domain motion, an event that is likely coupled to catalysis. We also demonstrate that the indole-3-carboxylic acid decarboxylase can be coupled with carboxylic acid reductase to produce indole-3-carboxyaldehyde from indole + CO_2_ under ambient conditions. These insights provide further evidence for a common mode of action in the widespread UbiD enzyme family.

Decarboxylases are involved in a wide range of biological processes and frequently depend on an organic cofactor (such as pyridoxal-5′-phosphate, biotin, thiamine diphosphate) or metal ions to facilitate carbon dioxide release. The UbiD decarboxylases are prevalent in microbes and reversibly decarboxylate (hetero)aromatic and unsaturated aliphatic acid compounds using the highly modified prenylated flavin mononucleotide (prFMN) cofactor (1). The latter is synthesized by the flavin prenyltransferase UbiX (2) and subsequently is proposed to undergo oxidative maturation within the context of the UbiD enzyme (3, 4). Crystal structures of several UbiD enzymes reveal the UbiD monomer is comprised of three domains: an N-terminal prFMN-binding domain connected *via* a long alpha-helical linker to the oligomerization domain, followed by a short C-terminal helix that associates with the prFMN-binding domain of an adjacent monomer. Domain motion of the prFMN-binding domain with respect to the oligomeric core of the enzyme links the open and closed states and is proposed to be an integral part of catalysis (5). As part of the generic UbiD reaction, the prFMN cofactor is proposed to undergo reversible covalent adduct formation with the substrate, facilitating the (de)carboxylative step. However, ligand complexes have proven elusive for most UbiDs, where crystal structures correspond to the open state of the enzyme. Indeed, ligand complexes have only been observed for the model system *Aspergillus niger* ferulic acid decarboxylase and the *Pseudomonas aeruginosa* pyrrole-2-carboxylate decarboxylase, where corresponding structures represent the closed state of the enzyme (6). Covalent catalysis by the prFMN-dependent UbiDs has been proposed to involve reversible 1,3-dipolar cycloaddition for alpha, beta-unsaturated acids such as ferulic, cinnamic, and p-coumaric acids. However, the UbiD enzyme family also operates on a wide range of (hetero)aromatic acids, with structurally characterized members including AroY (7), HmfF (8), vanillic acid decarboxylase (VdcCD) (5) and PA0245 (6) and a range of mechanisms have been proposed for prFMN adduct formation in these cases. Although UbiDs function as decarboxylases under physiological conditions, some anaerobic organisms are proposed to initiate degradation of recalcitrant aromatic compounds such as benzene or naphthalene *via* UbiD-mediated carboxylation (9, 10, 11). Indeed, coupling of the reversible decarboxylase reaction to an irreversible acid consuming step can yield C-H activation and CO_2_ fixation under ambient conditions (12). Alternatively, UbiD enzymes have been used as carboxylases *in vitro* in the presence of high [CO_2_] (13).

Here, we characterize I3C decarboxylase from *Arthrobacter nicotianae* (AnInD) (14). Indole is produced by a wide range of bacteria and can modify cellular redox state, facilitate anaerobic survival and biofilm formation, as well as act as a cell signal to regulate gene expression, spore formation, plasmid stability, drug resistance, and modify host cellular responses (15, 16). Several pathogens and chemical-inducible indolic metabolites have been reported from the leaves and roots of Arabidopsis, mainly indole-3-carboxylic acid and indole-3-carboxyaldehyde suggestive of roles of these metabolites in plant defense (17, 18). We provide structural and biochemical insights into AnInD, revealing the enzyme to be strictly specific for indole-3-carboxylic acid (I3C). Structural insights from crystal structure determination combined with small-angle X-ray scattering (SAXS) measurements reveal the enzyme undergoes an open-closed transition as a consequence of prFMN domain motion, an event likely coupled to catalysis. We also demonstrate AnInD can be coupled with carboxylic acid reductase in a one pot reaction to produce indole-3-carboxyaldehyde from indole + CO_2_ under ambient conditions. These insights provide further evidence for a common mode of action in the widespread UbiD enzyme family.

## Results

### Purification and reconstitution of AnInD

**Figure 1:**
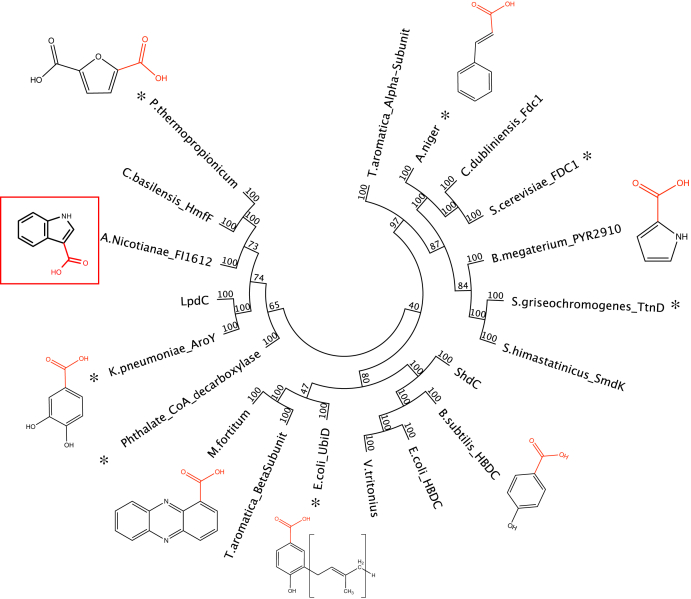
**Sequence alignment of AnInD with characterized UbiDs**. AnInD shares similarity with the HmfF cluster of the UbiD superfamily. All reported crystal structures of enzymes are indicated by ∗, and the substrates are shown next to enzymes characterized in previous studies. *Aspergillus niger* (XP_001390534), *Bacillus megaterium*_PYR2910 (WP_120038558), *Bacillus subtilis* VDC (WP_003246683), *Escherichia coli*_HBDC (WP_024253075), *Escherichia coli*_UbiD (HBB8332795), *Klebsiella pneumoniae*_AroY (XP_001390534), LpdC (AGE40339), ShdC (WP_179239558), *Aromatoleum* phthalate_coA_decarboxylase (WP_011255033), *Pelotomaculum thermopropionicum* (BAF58677), *Saccharomyces cerevisiae*_FDC1 (NP_010828), *Streptomyces griseochromogenes*_TtnD (6DA6_A), *Streptomyces himastatinicus*_SmdK (WP_009713959), *Cupriavidus basilensis*_HmfF (ADE20406), and *Candida dubliniensis*_Fdc1 (XP_002421128). AnInD, indole-3-carboxylate decarboxylase from Arthrobacter nicotianae.

**Figure 2:**
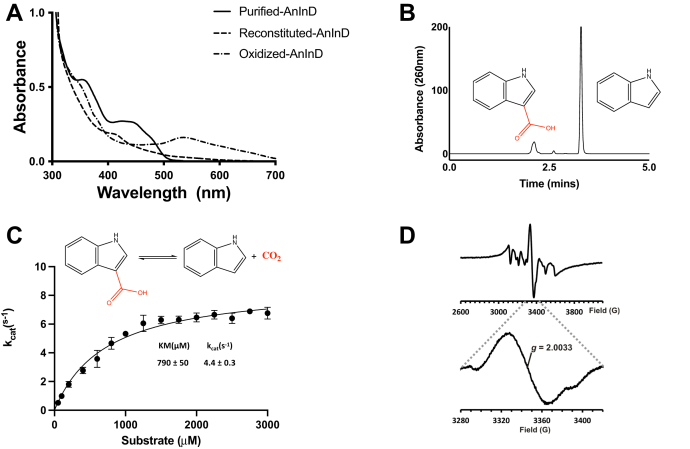
**Solution properties of purified AnInD**. *A*, UV-visible spectra of AnInD expressed without UbiX overexpression, after reconstitution with reduced prFMN and oxidation. Reconstituted AnInD^prFMN^ turns *purple* upon exposure to oxygen and develops a peak centered at ∼540 nm, indicative of prFMN^radical^ formation. *B*, HPLC chromatogram showing AnInD-mediated indole-3-carboxylic acid conversion to indole. *C*, kinetic parameters of AnInD determined by steady-state kinetics. *D*, EPR spectra of reconstituted AnInD. AnInD, indole-3-carboxylate decarboxylase from Arthrobacter nicotianae.

The amino acid sequence of AnInD was kindly provided by Professor Toyokazu Yoshida of Gifu University. Phylogenetic analysis of the AnInD sequence with characterized decarboxylases reveals that AnInD clusters with *Cupriavidus basilensis* HmfF (36% identity) and *Klebsiella pneumoniae* AroY (28% identity) (Fig. 1). HmfF acts on furan dicarboxylic acid while AroY affords 3,4-dihydroxybenzoic acid decarboxylation (7). In contrast, AnInD accepts the heteroaromatic I3C as a substrate (14). AnInD was heterologously expressed in *Escherichia coli* and purified to homogeneity by a single step purification procedure using Ni-nitriloacetic acid (Ni-NTA) agarose (Qiagen) affinity chromatography and appeared as a ∼50 kDa single band when analyzed by SDS-PAGE. Single-expressed AnInD (*i.e.*, in the absence of UbiX coexpression) was light yellow in color and displayed a UV-Vis spectrum similar to a mixture of oxidized flavin mononucleotide and prFMN, indicative of (weak) the binding of flavin and consistent with other reported UbiDs (3, 7, 8), (Fig. 2*A*). Following reconstitution with prFMN, the oxidized AnInD:prFMN complex develops a characteristic broad peak at ∼540 nm arising from the semiquinone radical prFMN (Fig. 2*A*). The X band continuous wave electron paramagnetic resonance (EPR) spectrum of the prFMN^radical^ lies at the center of the six line pattern arising from the ms = ½ manifold of the S = 5/2 Mn^2+^ ion, the six lines arising from hyperfine interaction with the I = 5/2 Mn nucleus (Fig. 2*D*). The EPR spectrum of AnInD^prFMN^ shows that prFMN is protein bound and is identical to the prFMN radical as observed for *A. niger* Fdc1, *E. coli* UbiD, and PA0245 (3, 6, 19).

### Oxidative maturation is required for AnInD decarboxylase activity

**Figure 3:**
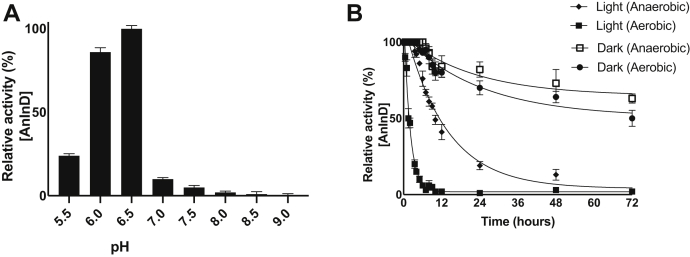
**AnInD oxygen and light sensitivity**. *A*, AnInD has an optimum pH of 6.5. *B*, AnInD decarboxylation activity diminishes rapidly upon exposure to light in aerobic conditions. AnInD half-life was calculated to be 1.63 ± 0.15 h in ambient light conditions. All assays were carried out at room temperature, and the protein samples were stored in the dark at 4 °C. AnInD, indole-3-carboxylate decarboxylase from Arthrobacter nicotianae.

The reduced prFMN supplied by UbiX must undergo oxidative maturation to yield the active prFMN^iminium^ form (2). Indole-3-carboxylic acid, decarboxylation can be observed spectrophotometrically at ∼260 nm. *Apo*-AnInD does not yield decarboxylation of I3C, however, reconstituted AnInD^prFMN^ exhibits weak activity with I3C under anaerobic conditions (Table S1). This is likely due to oxidative maturation by trace amounts of oxygen. In contrast, upon addition of the substrate in aerobic buffer, the activity of reconstituted AnInD^prFMN^ increases rapidly (Fig. 2, *B* and *C* and Table S1). The decarboxylase activity was confirmed by HPLC analysis and shows that AnInD requires oxidative maturation to attain catalytic efficiency. Screening of the effect of pH on AnInD^prFMN^ decarboxylase activity reveals a bell-shaped response with an optimal pH for I3C decarboxylase activity of 6.5 and a corresponding k_cat_ of 4.3 ± 0.6 s^−1^ (mean ± SD, n = 3) with a 50% loss of specific activity at pH 5.7 and pH 6.8 (Fig. 3*A*). The Michaelis–Menten kinetics for I3C decarboxylation yielded K_m_^app^ and k_cat_^app^ values of 790 μM and 4.4 (±0.3) s^−1^, respectively (Fig. 2*C*). These are presented as apparent values due to the presence of a minor inactive prFMN radical species that complicates accurate quantification of active enzyme concentration.

### AnInD is light and oxygen sensitive

Several UbiD family members have been reported to be light and/or oxygen sensitive and prone to lose activity over time (3, 8). We therefore sought to investigate the stability of AnInD in the presence of light and oxygen. The half-life for AnInD decarboxylation activity under anaerobic conditions and ambient light was determined to be 9 ± 0.45 h (Fig. 3*B*). When AnInD is stored in the dark under anaerobic conditions, activity remains stable for several hours, with 40% activity loss after 72h. In contrast, the decarboxylase activity of AnInD^prFMN^ decreases rapidly when stored aerobically (under normal illumination) with a half-life of approximately 1.6 ± 0.2 h. Similarly, when stored aerobically in the dark, AnInD retains activity better as observed by spectrophotometric assays (Fig. 3*B*). The fact that AnInD is light sensitive is similar to that observed with *A. niger* Fdc1, where cofactor tautomerized upon light exposure leading to enzyme inactivation (19). However, while Fdc1 is not reported to be sensitive to oxygen, O_2_ sensitivity has been reported for other UbiDs such as PtHmfF and AroY (3, 8).

### AnInD mediates indole-3-carboxylation and H/D exchange

**Figure 4:**
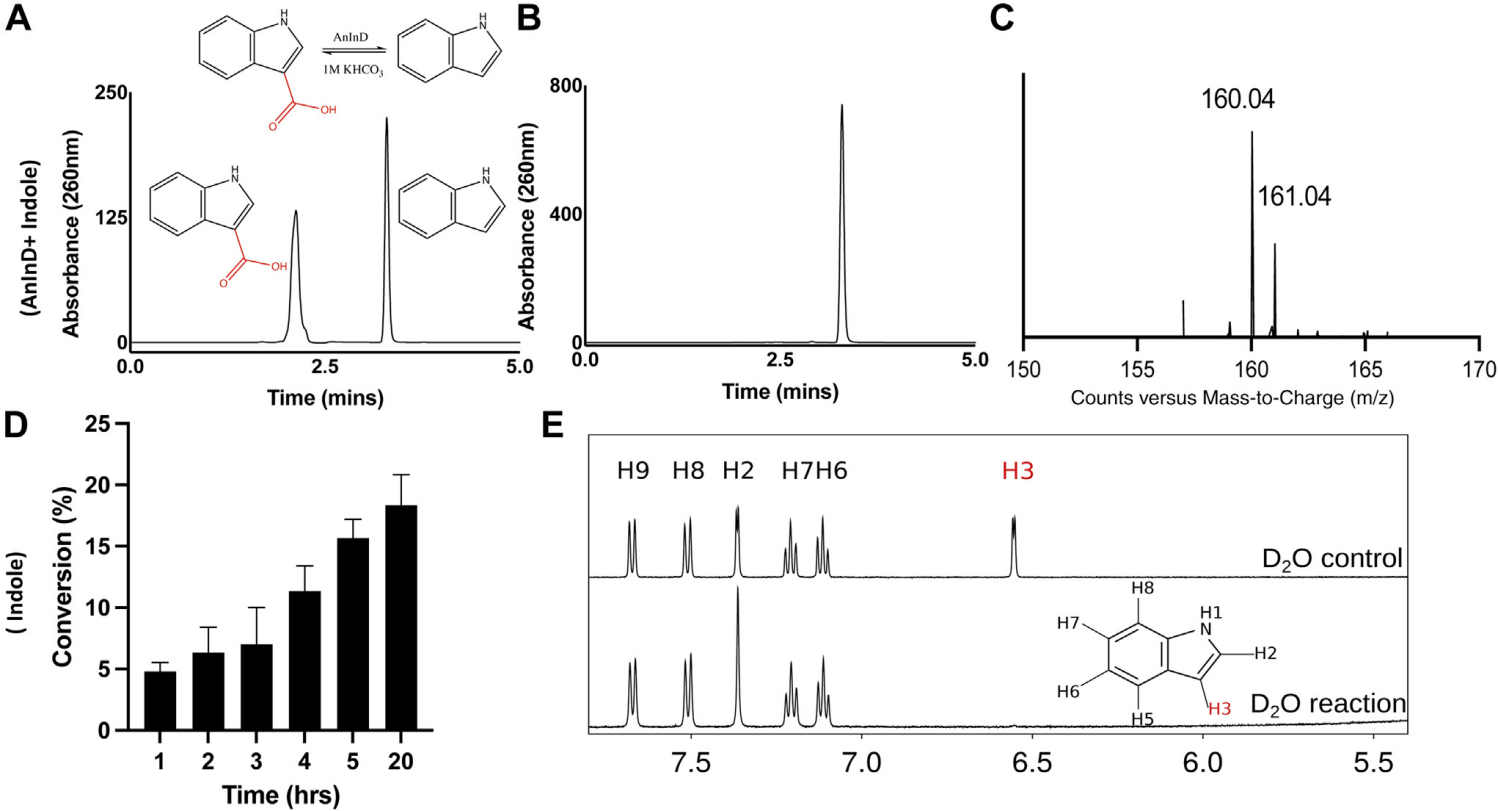
**Indole carboxylation reaction by AnInD in the presence of 1 M KHCO**_**3**_**.***A*, HPLC chromatogram showing carboxylation reaction of indole by AnInD. Both indole and indole-3-carboxylic acid were observed in the chromatogram. *B*, no conversion/carboxylation was detected in case of negative control. *C*, mass spectrometry chromatograms showing C^13^ exchange in the presence of 1 M NaHC^13^O_3_. *D*, time-dependent conversion of indole to indole-3-carboxylic acid by AnInD shows maximum conversion of 18% following overnight incubations under the assay conditions used. *E*, 1H NMR showing proton exchange of indole in the presence of AnInD in deuterated buffer (D_2_O). The H3 peak disappears in the presence of AnInD. AnInD, indole-3-carboxylate decarboxylase from Arthrobacter nicotianae.

AnInD has been previously reported to catalyze indole carboxylation in the presence of high CO_2_ concentration (14). We investigated the carboxylation of indole with the purified coexpressed/reconstituted AnInD^prFMN^ protein in the presence of NaHCO_3_ as a source of CO_2_. Reactions were incubated overnight at 30 °C in tightly sealed glass vials, and the samples were prepared and analyzed by HPLC. The conversion of indole to I3C was observed after 12 h incubation with a maximum yield of ∼18 % (Fig. 4, *A* and *D*). No I3C was observed in negative control reactions (Fig. 4*B*). We also observed ^13^C enrichment by AnInD^prFMN^ of I3C in the presence of 100 mM NaH^13^CO_3_ (Fig. 4*C*). Similarly, ^1^H NMR showed that the incubation of indole with AnInD^prFMN^ in D_2_O resulted in the disappearance of the indole H3 resonance peak, which indicates H/D exchange at the H3 position (Fig. 4*E*).

### One pot reaction of AnInD and *Seginiliparous rugosus* carboxylic acid reductase to produce indole-3-carboxyaldehyde

**Figure 5:**
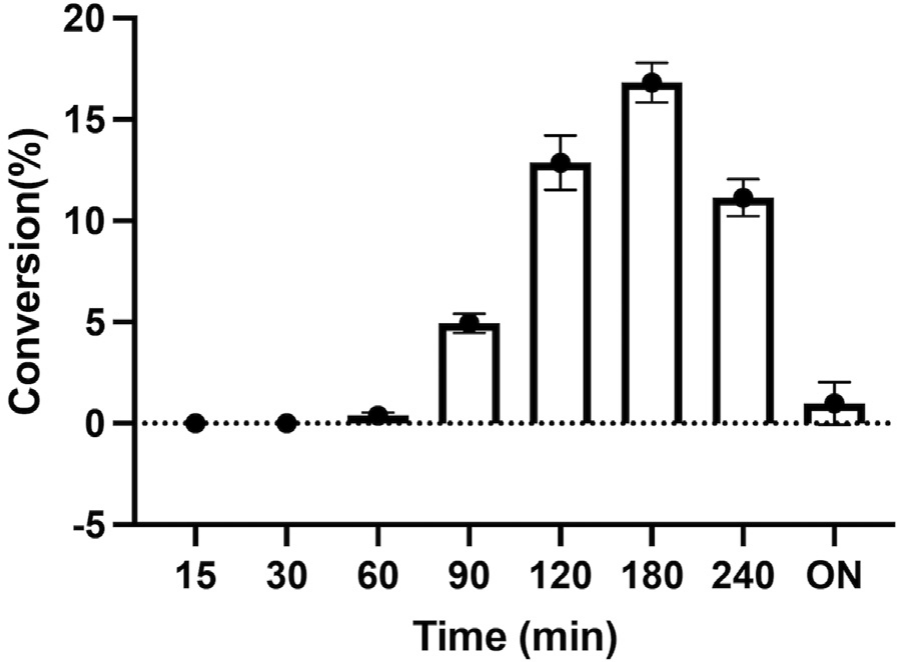
**Coupling of AnInD with SrCAR yields indole-3-carboxyaldehyde.** Assays contain 5 μM AnInD, 5 mM indole, 0.25 M NaHCO_3_, 4 mM ATP, 3 mM NADPH, and 2 μM SrCAR in 50 mM Kpi pH 6.5. Under the conditions used, maximum yield (∼16%) of the product indole-3-carboxyaldehyde was observed after 3h. AnInD, indole-3-carboxylate decarboxylase from Arthrobacter nicotianae; SrCAR, *Seginiliparous rugosus* carboxylic acid reductase.

We investigated the coupling of AnInD with carboxylic acid reductases (CARs) to push the equilibrium in favor of the formation of indole-3-carboxyaldehyde, a pharmaceutically important product. To produce a high yield conversion of indole to indole-3-carboxyaldehyde, purified AnInD^prFMN^ and SrCAR (*Seginiliparous rugosus* CAR) enzymes were assembled in a one pot reaction, and the rate of conversion was monitored in the presence of different concentrations of NaHCO_3_ at a final pH 7.0 and 8.0 (Fig. S1). These assays revealed the formation/production of indole-3-carboxyaldehyde. The optimum condition for maximum conversion (∼16%) was obtained with 0.25 M NaHCO_3_ at final pH 7.0 (Fig. 5). The inherent reactivity of the CAR aldehyde product likely precludes accumulation over time to high yields but offers further opportunities to extend the application of this cascade by coupling with other industrially relevant enzymes.

### AnInD substrate range is limited to I3C

We investigated the substrate scope of AnInD^prFMN^ against a range of (hetero)aromatic compounds. No evidence of decarboxylase activity could be observed for indole-2-carboxylic acid, pyrrole-2-carboxylic acid, indazole-3-carboxylic acid, quinoxaline-2-carboxylic acid, quinoline-2-carboxylic acid, indene-3-carboxylic acid, benzothiophene-3-carboxylic acid, benzothiophene-2-carboxylic acid, 2-naphthoic acid, and 1-naphthoic acid either by spectrophotometric or HPLC analysis. Hence, AnInD^prFMN^ exhibits strict substrate specificity for I3C only.

### AnInD crystal structure determination

**Figure 6:**
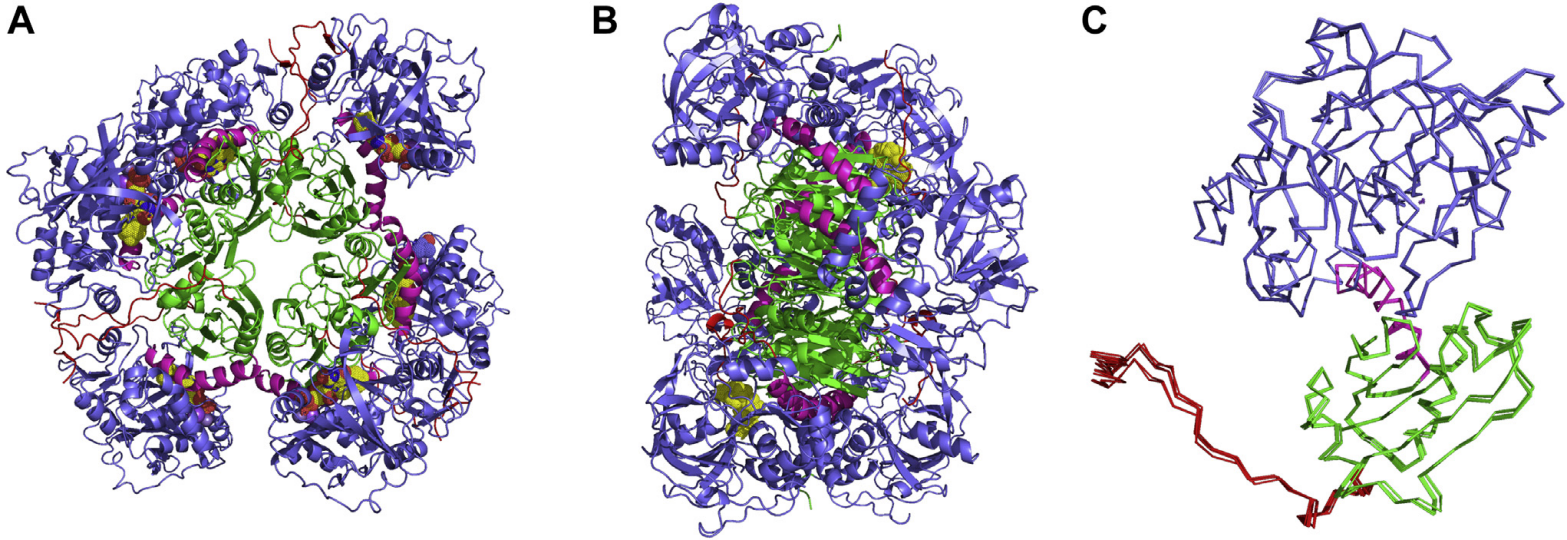
**Crystal structure of AnInD.***A*, and *B*, AnInD crystal structure depicted by *cartoon* representation (in two orientations) with the prFMN-binding domain in *blue*, connecting domain in *magenta*, dimerization domain in *green*, and the C-terminal tail in *red*. *C*, superposition of the six AnInD monomers present in the asymmetric unit. AnInD, indole-3-carboxylate decarboxylase from Arthrobacter nicotianae; prFMN, prenylated flavin mononucleotide.

**Figure 7:**
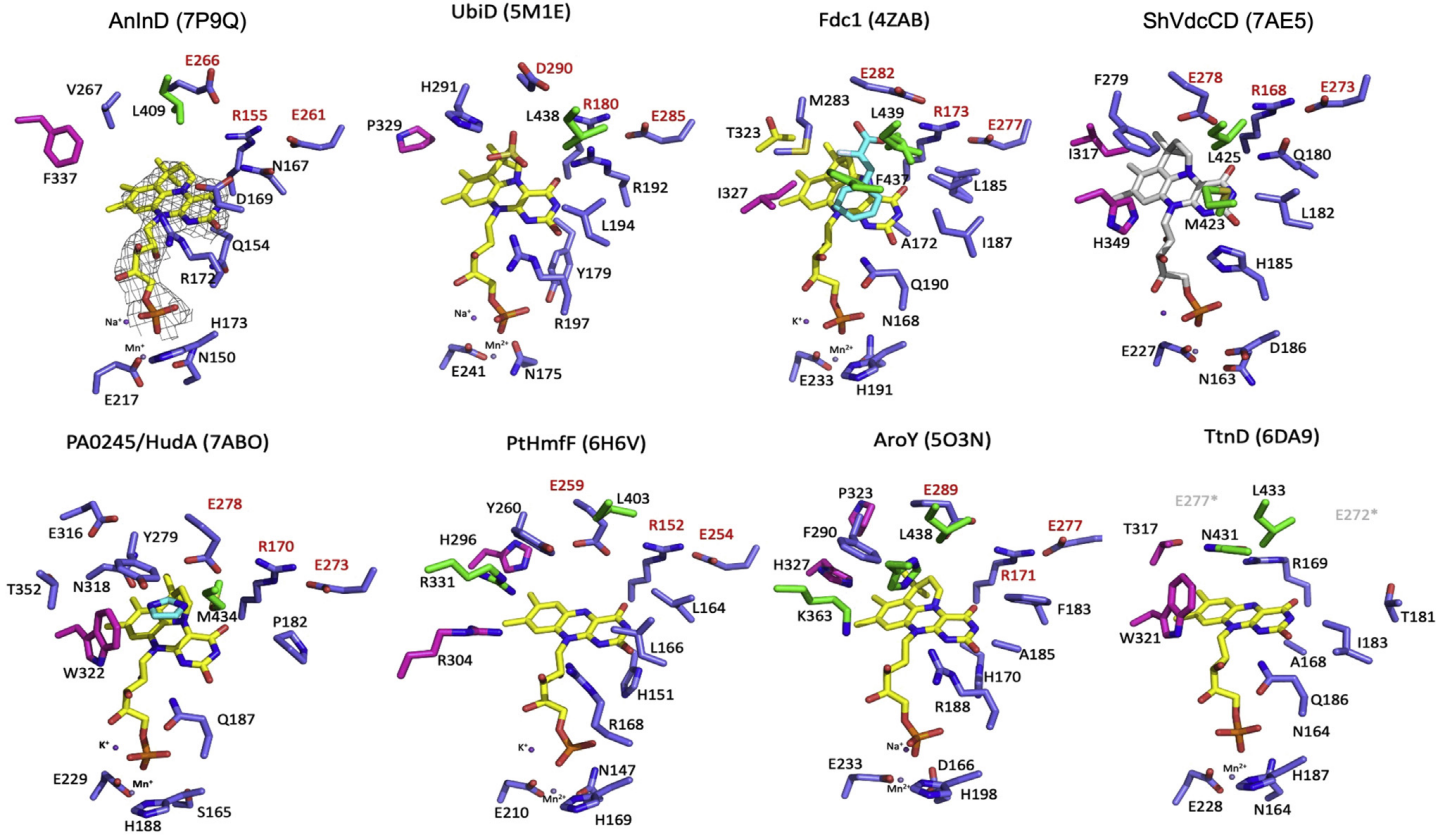
**Active site of AnInD compared to other UbiD family members.** Fdc1 (4ZAB), AroY (5O3N), PtHmfF (6H6V)*, Escherichia coli* UbiD (5M1E), TtnD (6DA9), ShVdcCD (7AE5), and PA0245 (7ABO). The bound-prFMN cofactor is shown in *yellow sticks*. In the case of VdcCD, prFMN was modeled into the active site by superposition and is shown in *gray sticks*. All the amino acids shown are colored according to the domain structure as depicted in Fig. 6. The conserved residue triad E(D)-R-E is highlighted with *red labeling*. AnInD, indole-3-carboxylate decarboxylase from Arthrobacter nicotianae; prFMN, prenylated flavin mononucleotide; VdcCD, vanillic acid decarboxylase.

We sought to determine the crystal structure of AnInD to rationalize the strict substrate specificity. Diffraction quality crystals were obtained from overnight crystallization trials. Crystals diffracted to 2.2 Å resolution and belong to space group P2_1_. AnInD structure in complex with prFMN was solved by molecular replacement. Data collection and refinement statistics are summarized in Table 1. The AnInD structure contains an entire hexamer in the asymmetric unit (Fig. 6, *A* and *B*). Each monomer of AnInD is composed of an N-terminal prFMN-binding domain connected to the oligomerization domain *via* an alpha helical linker. The C-terminal extended loop projects out and forms interactions with the prFMN-binding domain of the adjacent monomer (Fig. 6*C*). The AnInD monomer has a Z score of 47 with PtHmfF (PDB id: 6H6V, r.m.s.d: 1.8 Å for 400 *C-αs*), 42.7 with *E. coli* UbiD (PDB id: 5NY5 r.m.sd: 1.7 Å for 338 *C-αs*), and 42.4 with AroY (PDB id: 5O3M, r.m.sd: 1.7 Å for 335 *C-αs*). The cofactor complex structure was obtained after soaking AnInD crystals with freeze-dried prFMN powder (20). An overlay of the individual AnInD monomers reveals minor variations in the respective positions of the prFMN-binding domain and oligomerization domain. Electron density of bound prFMN and associated metal ions could be readily observed in the active site of the AnInD–prFMN complex structure. The phosphate moiety of the prFMN is coordinated by two metal ions, identified as Mn^2+^ and Na^+^ as these were present in the sample and by analogy to other UbiD enzymes. The prFMN-modified isoalloxazine ring is placed adjacent to the conserved E(D)-R-E network of ionic residues that is conserved in the UbiD family (Figs. 7 and S2). The relatively large distance between the conserved residues Leu409 and Arg155 (∼12.66 Å for the respective center of mass) indicates the AnInD crystal structure corresponds to an open conformation as observed for AroY (PDB id: 5O3M), *E. coli* UbiD (PDB id: 5NY5) and HmfF (PDB id: 6H6V), and the open VdcCD (PDB id: 7AE5). A potential hinge region located on a central alpha helix (G310) can be identified, which connects the prFMN-binding domain and oligomerization domain (Fig. 6*C*). A similar hinge position has been reported in the case of ShVdcCD (5). We have attempted to obtain structural data of AnInD in complex with the substrate but neither cocrystallization nor soaking experiments have proven successful. This is likely due to the open conformation of the AnInD structure and unfortunately, crystals corresponding to the closed state could not be obtained.

**Table 1:** Summary of data collection and structure refinement statistics

Parameters	prFMN-AnInD (PDB id: 7P9Q)
Wavelength (λ)	0.9762
Resolution range	43.29–2.53 (2.62–2.53)
Space group	P1211
Unit cells	
a, b, c (Å)	104.2, 195.45, 105.21
a, b, g (°)	90,116.35, 90
Total reflection	123968 (12473)
Unique reflection	6164 (617)
Multiplicity	3.4 (3.5)
Completeness	98.9(99.6)
I/sI	9.7 (0.9)
R_merge_	0.079(0.028)
R-meas	0.051(0.018)
CC1/2	1.0(0.4)
Reflection used in refinement	123902(12472)
Reflection used for R-free	1017(95)
R_work_	0.1871 (0.3521)
R_free_	0.2385 (0.4141)
No. of nonhydrogen atoms	21150
Macromolecules	20814
Ligand	228
Solvent	108
Protein residues	2742
RMS(bonds)	0.015
RMS (angles)	1.84
Ramachandran favored (%)	94.81
Ramachandran allowed (%)	4.34
Ramachandran outlier (%)	0.85
Rotamer outlier (%)	0.05
Clashscore	19.07
Average B-factor	101.14
Macromolecules	101.18
Ligand	112.88
Solvent	69.17
Number of TLS group	22

Each structure was determined from one crystal.

∗Values in parentheses are for highest-resolution shell.

### Model building of the closed conformation

Superposition of the AnInD structure with the Fdc1 (PDB id: 6TIB), PA0245 (PDB id: 7ABO), and VdcCD (PDB id: 7AE4) structure enables us to construct a model of the closed AnInD active site. The closed structure of VdcCD (PDB id: 7AE4) was used as a reference model for generation of the AnInD closed model. In the VdcCD closed structure, a point of rotation of oligomerization domain was identified between T325 and S326. Superposition of the prFMN-binding domain of AnInD on the closed VdcCD structure enabled us to identify a corresponding hinge region (G310) on the helix, which connects the prFMN-binding domain and the oligomerization domain. A rigid body superposition of the oligomerization domain of AnInD on to the closed VdcCD oligomerization domain allowed us to produce an initial closed AnInD model (Fig. S3). Energy minimization of the obtained closed AnInD model was done in ICM-Pro. Average R-L distance of the closed model was 11.12 Å calculated by CALCOM server (21).

### Small-angle X-ray scattering reveals that AnInD likely explores open and closed states in solution

**Figure 8:**
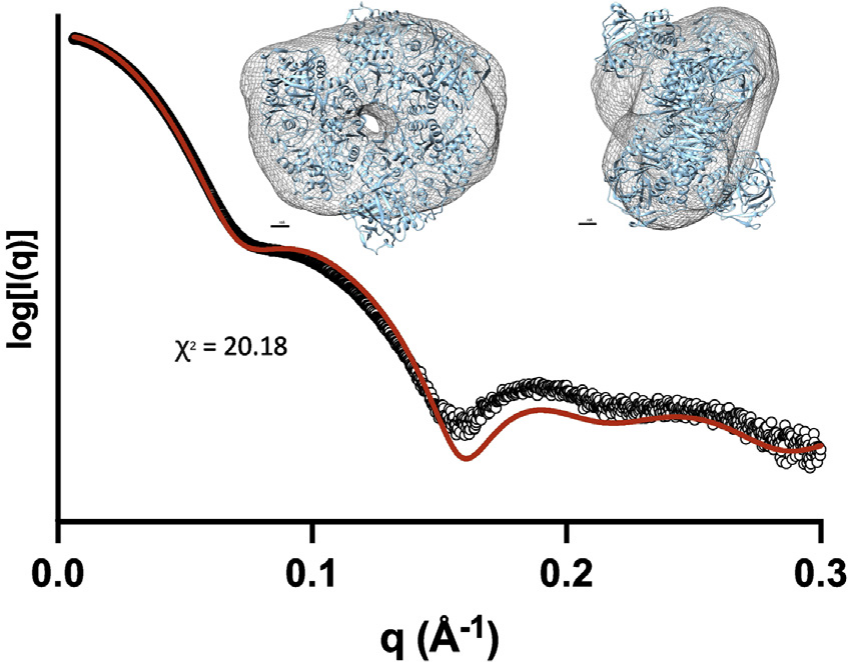
**Analysis of AnInD by small-angle X-ray Scattering.** Plots of X-ray scattering intensity log(I(q)) as a function of the scattering vector q (Å^−1^) for AnInD. **Small angle X-ray scattering** data are shown as *black circles*, and the normalized fit from the best individual molecular model is shown as a *red line* (calculated with FoXS, goodness of fit indicated with its χ^2^ value). (Inset) *Ab initio* reconstructions calculated with DAMAVER are shown as *transparent mesh envelope* with the hexamer crystal structure of AnInD superimposed using the automatic map fitting tool implemented in Chimera. AnInD, indole-3-carboxylate decarboxylase from Arthrobacter nicotianae.

Recently, crystal structures of the UbiD enzymes PA0245 and VdcCD were reported for both the open and closed conformations, suggesting domain motion dynamics underpins the (de)carboxylation activity with (hetero)aromatic substrates (5, 6). We have used SAXS to investigate potential domain motion for AnInD. Scattering profiles of all the data sets indicate the data was of sufficient quality to pursue further analysis. Analysis of dimensionless Kratky plots showed that the enzyme is a folded globular protein as suggested by the shape of the curves and the peak maxima coincide with the globularity point. Porod exponents also indicate the compactness of the protein in solution (Fig. S4). Estimates of the radius of gyration (R_g_) for each dataset were calculated from the Guinier region-using primus, and maximum dimension (D_max_) was obtained from indirect Fourier transformation of the scattering profiles using GNOM (Table 2). Particle shape of the enzyme was restored by scattering profile using DAMMIN (22). The envelope generated revealed the hexameric nature of AnInD (Fig. 8) similar to other UbiD family members (5, 7). Furthermore, we tried to model the scattering data using a range of AnInD models, including fully open, fully closed, and intermediate species (Fig. S5). There are no clashes observed between individual domains respectively in the open and closed positions (*i.e.*, the intermediate species), indicating that any communication between active sites (if indeed this occurs) is unlikely to rely on simple steric hindrance. The scattering profile predicted from the hexamer crystal open structure and partial open/closed models capture many of the essential features of the experimental scattering. However, it falls below the data at mid-q (0.07–0.15 Å^−1^), giving a sub-optimal fit (Fig. 8). Theoretical solution scattering of the individual models tested did not fit with the experimental scattering, likely indicating that the AnInD hexamer can adopt a wide range of different conformations in solution.

**Table 2:** Summary of biophysical parameters of AnInD

Biophysical parameters	AnInD	
	SAXS	Crystal (PDB id: 7P9Q)
Rg (Å)	47.38	46.80
Dmax(Å)	132.5	145.5

### Docking of the I3C provides a rationale for substrate specificity

**Figure 9:**
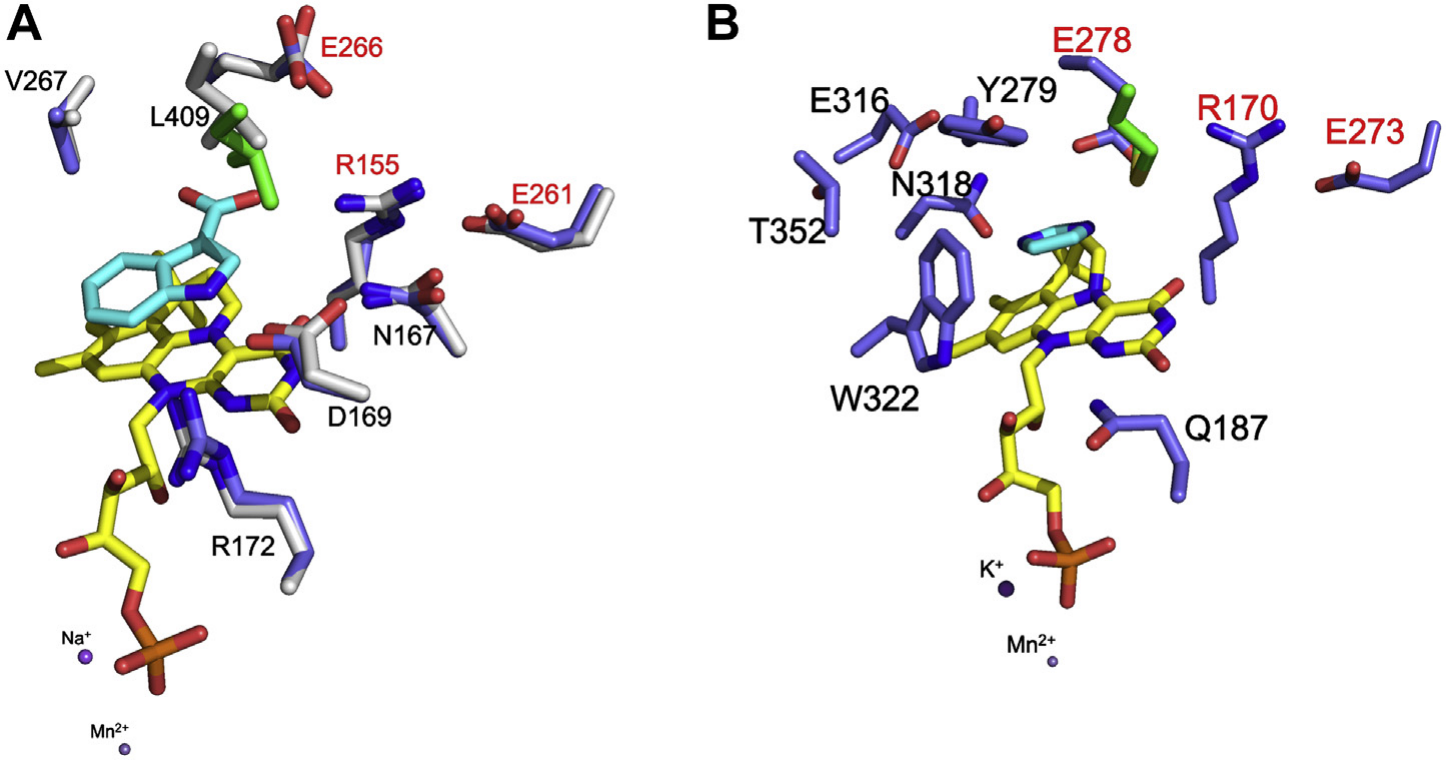
**A model for the indole-3-carboxylic acid AnInD complex.***A*, closed model of AnInD was built on the closed crystal structure of VdcD, and substrate indole-3-carboxylic acid was docked in the active site using ICM-pro. Active site residues of the open crystal structure are shown in gray color, and the corresponding closed conformations are shown in the same color as Fig. 7. *B*, PA0245 imidazole complex structure (PDB: 7ABO). Active site residues are displayed as *blue sticks* in the closed model and *gray* in open crystal structure, prFMN cofactor in *yellow* and substrates are shown in *cyan*. AnInD, indole-3-carboxylate decarboxylase from Arthrobacter nicotianae; prFMN, prenylated flavin mononucleotide; VdcCD, vanillic acid decarboxylase.

**Figure 10:**
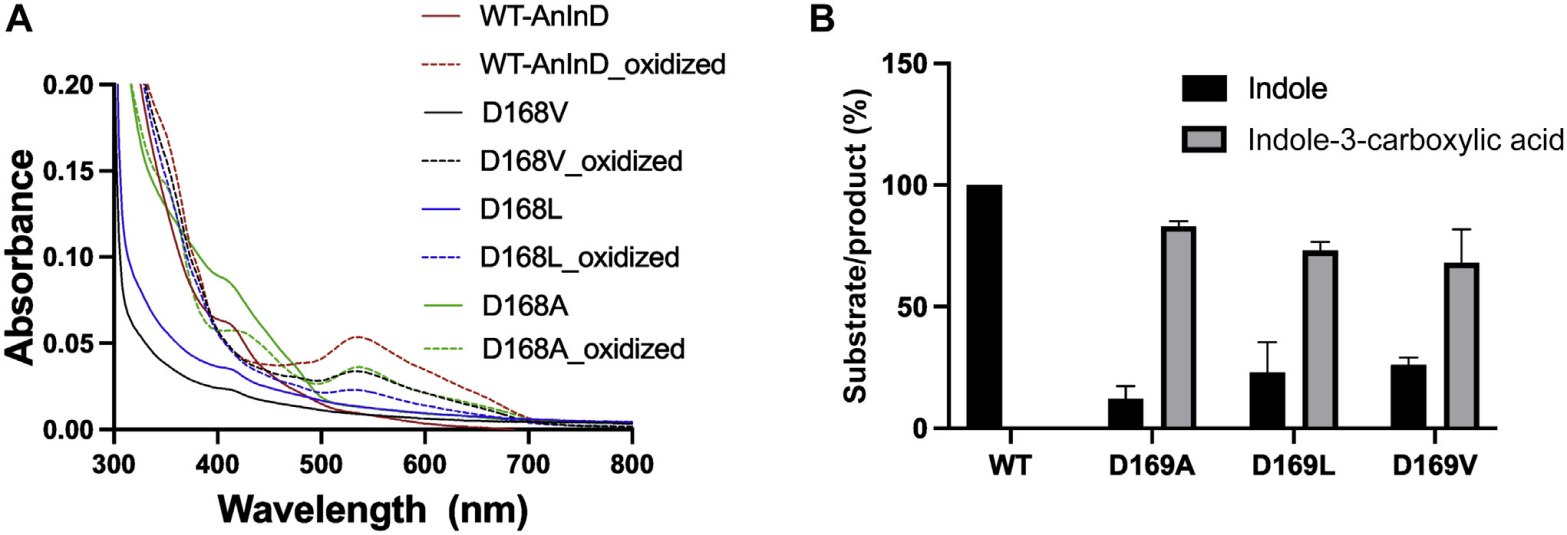
**Characterization of AnInD variants.***A*, UV-Vis spectra of reconstituted and oxidized AnInD variants, zoomed on prFMN-related features. *B*, end point HPLC assays of AnInD^prFMN^ WT and D169 variants with indole-3-carboxylic acid, either for substrate depletion or product formation. AnInD, indole-3-carboxylate decarboxylase from Arthrobacter nicotianae; prFMN, prenylated flavin mononucleotide.

In the absence of experimental AnInD ligand complex structures, we sought to model the enzyme substrate complex *via* docking into open and closed structures. The catalytic glutamate residue (E266) adopts a range of conformations as previously reported for other UbiD enzymes (19) due to the type of ligands/substrates bound in the active site, hence the rotamer of E266 was changed in order to avoid potential I3C clashing during docking. The substrate was docked into the active site of the open crystal structure and closed AnInD model using a docking algorithm implemented in ICM-Pro. In both open and closed state docked models, I3C binds in a similar position in the AnInD active site, however, the binding score was higher in the case of the closed model (RTCNN score: −30.02) as compared to the open crystal structure (−19.5), where other noncatalytically relevant modes of binding scored higher. The I3C docking reveals the substrate indole nitrogen is within hydrogen bonding distance to D169, while the I3C carboxylic acid moiety interacts with the R155 sidechain and E266 backbone (Fig. 9*A*), both of which are conserved in the UbiD family (Fig. S2) (19). To support our hypothesis regarding the role of D169 in substrate binding, we made D169V, D169L, and D169A variants of AnInD. Following purification and reconstituted with prFMN, individual variants showed distinct levels of prFMN incorporation as observed by UV-Vis spectrophotometry (Fig. 10*A*). All AnInD D169X variants were found to be severely affected in activity as compared to the WT enzyme (Fig. 10*B*), suggesting an important role for D169 in substrate binding.

## Discussion

**Figure 11:**
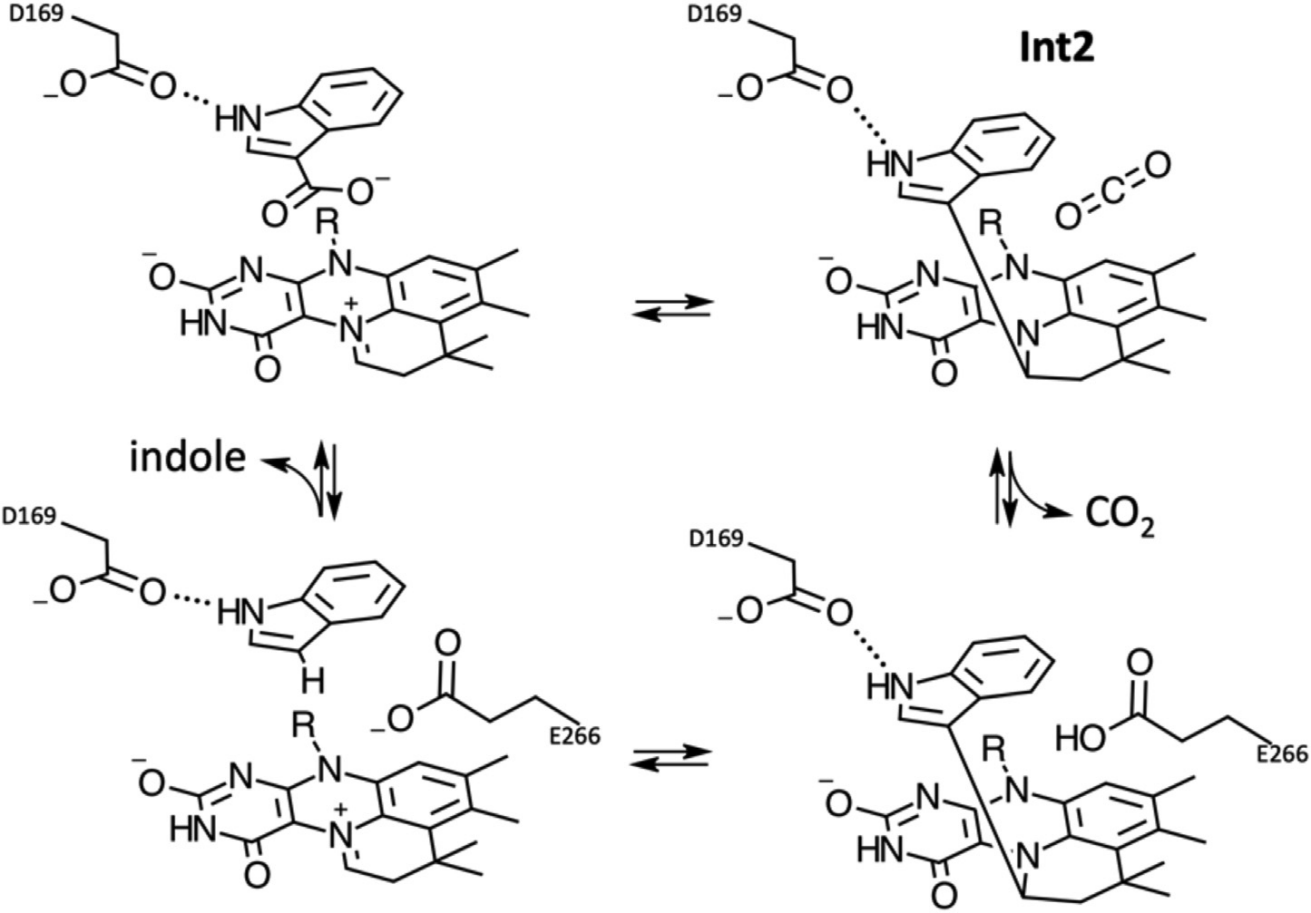
**Proposed mechanism of AnInD decarboxylation.** Reversible decarboxylation is mediated through prFMN covalent catalysis. Similar to other proposed UbiD mechanisms, this is likely to proceed through a range of intermediates, with the aromatic indole-prFMN **IntII** adduct likely formed concomitant with domain motion. The nature of **IntI** and **IntIII** species preceding or following from **IntII** depends on whether a 1,3 dipolar cycloaddition or electrophilic addition mechanism applies in this case. AnInD, indole-3-carboxylate decarboxylase from Arthrobacter nicotianae; prFMN, prenylated flavin mononucleotide.

Recent studies have shown that UbiD enzymes can be used for (hetero)aromatic C-H activation at ambient conditions and can provide a sustainable and green way to corresponding acids and derivative compounds (8, 12, 23). The production of *cis,cis*-muconic acid (24, 25) and 1,3-butadiene (26), which are valuable chemicals for synthetic polymer production has already been shown. The processing and utilization of biomass derived (hetero)aromatic compounds can lead to a reduce dependence on oil-based compounds (27, 28, 29, 30). The covalent catalysis used by the UbiD enzymes presents a range of challenges to ensure rapid turnover. In the case of the model enzyme *A. niger* Fdc1, considerable strain is imposed on covalent adducts made between cinnamic acid and the cofactor (19). This ensures that a reversible 1,3-dipolar cycloaddition process takes place. However, the UbiD family’s substrate scope extends beyond cinnamic-acid and includes heteroatomic and aromatic acid compounds (1). With (hetero)aromatic substrates, the aromatic nature of the covalent adducts presents additional challenges. A large motion of the substrate aromatic moiety likely occurs as the reaction progresses. Recently, VdcCD open and closed crystal structures and modeling studies demonstrated that domain motion is likely coupled to catalysis (5). The crystal structure of AnInD remains poised toward the open conformation as frequently observed for other UbiDs (3, 7, 8). The experimental scattering profile suggests that the enzyme adopts a dynamic equilibrium in solution, and a closed conformation can be generated on the basis of homology with the closed VdcCD structure. This allows modeling of the enzyme-substrate complex and reveals D169 as a likely H-bonding partner for the substrate indole group. The substrate alpha-beta unsaturated bond is placed directly above the prFMN^iminium^ C1′-N5-C4a, similar to other UbiD-ligand complexes. Formation of the covalent aromatic adduct **Int2** following decarboxylation is either preceded by a 1,3 cycloaddition process or an electrophilic addition assisted by acid-base catalysis of D169 (Fig. 11). Mutagenesis of the latter drastically affects activity, although it remains unclear to what extent this is linked to affects on substrate binding or catalysis. The AnInD enzyme is unable to accept other structurally related substrates, suggesting the indole moiety plays a key role too. Further studies will be required to fully elucidate the nature of steps preceding the (de)carboxylation event.

At ambient conditions, the UbiD reaction is poised toward decarboxylation. However, it has recently been shown that UbiD-cascade reaction systems have the potential to activate unsaturated C*sp*^*2*^-H substrates to a range of C + 1 compounds concomitant with CO_2_ fixation (12). We here demonstrate the principle applies to non-Fdc1 UbiD enzymes by coupling carboxylic acid reductase with AnInD, leading to the formation of indole-3-carboxyaldehyde from indole in one pot synthesis. Indole-3-carboxyaldehyde is a pharmaceutically relevant molecule and has been shown to be important in the synthesis of various analogs of anticancer molecules such as indole phytoalexins brassinin and 1-methoxyspirobrassinol methyl ether (31).

## Experimental procedures

### Cloning and mutagenesis

The *E. coli* codon optimized I3C UbiD gene from *A. nicotianae* was synthesized by GeneArt (Thermo-Fisher). Polymerase chain reaction was performed with Phusion polymerase (NEB). The genes were subcloned into pET30a and pET28a expression vectors with a C-terminal and N-terminal hexahistidine tag respectively using In-Fusion ligation independent cloning (Clontech). DNA construct sequences were confirmed (Eurofins Genomics sequencing) and the purified plasmid transformed into *E. coli* BL21(DE3) for protein expression (NEB). To coexpress decarboxylases with UbiX, BL21(DE3) cells were cotransformed with *P. aeruginosa* UbiX (pCDF-UbiX) together with the decarboxylase plasmid. *S. rugosus* carboxylic acid reductase was cloned as described previously (32). Site-specific changes in WT constructs were introduced by the quick-change method and once the presence of the desired mutation was confirmed by DNA sequencing, the plasmid was transformed into *E. coli* BL21(DE3)

### Protein expression and purification of hexahistidine tagged decarboxylases

Proteins were over expressed in *E. coli* BL21(DE3) cells grown at 37 °C either in Terrific Broth media with induction by 0.25 mM IPTG overnight at 20 °C or in Terrific Broth Auto Induction media (Formedium) at 24 °C for 48 h. All anaerobic purification steps were carried out in an anaerobic chamber (Belle Technology), operating at < 1 ppm oxygen and 18 °C. The cells were resuspended in anaerobic 50 mM Tris pH 7.5, 200 mM NaCl, 10% glycerol (buffer A) containing DNase, RNase, and SigmaFast EDTA free protease inhibitor cocktail (Sigma). Cells were lysed by passage through a French pressure cell at 17.5 Kpsi with sample and collection bottles under a constant flow of nitrogen gas. Cell lysates were clarified by ultracentrifugation at 185,000*g* for 1 h at 4 °C. The supernatant was applied to a 5 ml Ni-NTA agarose gravity flow column (Qiagen) in the anaerobic chamber. The resin was washed with buffer A followed by additional wash steps with buffer A containing 10 and 40 mM imidazole. Protein was eluted using buffer A containing 200 mM imidazole. Samples from the lysate and wash and elution fractions were analyzed by 4 to 20 % SDS-PAGE to determine fractions containing the protein of interest, and imidazole was removed from these fractions using a PD10 desalting resin (Bio-Rad). Purified protein was flash frozen in bead form and stored in liquid nitrogen for further experimentation. For purification of AnInD on the bench, all the steps were performed aerobically but in the dark and at 4 °C in the cold room. Protein was purified using the same procedure with the exception cells were lysed using a cell disruptor (Constant Cell Disruption Systems) at 20 Kpsi. Purified AnInD fractions from the Ni-NTA column were pooled and buffer exchanged with buffer A by passage down a PD10 desalting column. Purified AnInD protein was concentrated in a Vivaspin 10 kDa cut off spin concentrator, flash frozen in liquid nitrogen, and stored at −80 °C until further use. *S. rugosus* carboxylic acid reductase protein was purified as described previously (32).

### UV-visible spectroscopy and protein quantification

UV-visible absorbance spectra were recorded using a Cary 50 Bio spectrophotometer (Varian). Protein concentrations were estimated from the A_280_ absorption peak using extinction coefficients of AnInD (ε_280_ = 38,515 M^−1^ cm^−1^). Extinction coefficients were calculated from the primary amino acid sequences using the ExPASy ProtParam proteomics server. All spectra have been normalized for protein content.

### *In vitro* prFMN synthesis and enzyme reconstitution

A typical prFMN production reaction, containing 1 mM FMN, 2 mM DMAP, 5 mM NADH, 50 μM Fre reductase (purified as described by Shepherd *et al* 2015), and 50 μM UbiX in 50 mM Tris pH 7.5, 200 mM NaCl, was incubated at room temperature for a minimum of 3 h in an anaerobic glove box operating under 100% N_2_ (Belle Technology). The reaction mixture was filtered through a 10k MWCO spin concentrator (Vivaspin) to remove UbiX and Fre reductase proteins from the reaction mixture. Filtered prFMN cofactor mix was added to apodecarboxylase proteins in the presence of 1 mM MnCl_2_ in a molar ratio of 2:1 and incubated for 10 min. Excess prFMN cofactor was removed by passage through a PD10 desalting column (GE Healthcare) equilibrated in buffer A plus 1 mM MnCl_2_. Spectral features of reconstituted proteins were recorded by UV-Vis spectroscopy using a Cary 50 Bio spectrophotometer (Varian).

### Enzyme kinetics and substrates screening

Initial rates of substrate consumption were determined by monitoring the linear decrease in absorbance at the substrate λ_max_ using a Cary 50 Bio spectrophotometer. AnInD assays were performed against various concentrations of I3C substrate in 50 mM potassium phosphate pH 6.5 containing 50 mM KCl at room temperature. The rate of I3C decarboxylation was determined using A_270nm_ and the extinction coefficient of I3C (ε_270_ = 1051 M^−1^ cm^−1^). All the values are apparent because the prFMN content is unknown. AnInD proteins used for light and dark activity analysis were stored either in a black Eppendorf tube at 4 °C or light exposed in a clear Eppendorf tube at 4 °C.

### High-pressure liquid chromatography assays

Assays were performed in 50 mM potassium phosphate buffer pH 6.5 with 10 mM I3C and 2 μM AnInD enzyme. Reactions were quenched by the addition of an equal volume of acetonitrile containing 0.1% TFA and centrifuged at 16,100*g* to remove precipitate. Sample analysis was performed using a 1260 Infinity Series HPLC. The stationary phase was a Kinetex 5 μm C18 100 Å column, 250 × 4.6 mm and the mobile phase was acetonitrile: H_2_O (70:30) (v/v) with 0.1% TFA at a flow rate of 1 ml min^−1^. Detection of I3C and indole was done at 260 nM. The optimal pH for enzyme activity was investigated using the SPG three buffer-system (Qiagen). Mixing different ratios of succinic acid, sodium dihydrogen phosphate, and glycine attained pH values over the range 5.5 to 9. Where necessary, buffer solutions were made anaerobic by sparging with nitrogen gas before use.

### Indole carboxylation assays

Carboxylation reactions were performed in buffer 50 mM potassium phosphate pH 7.5, 150 mM KCl, 1 M NaHCO_3_ in 2 ml amber crimp seal vials. One to ten millimolar indole and 2 μM purified AnInD protein were mixed in a total volume of 500 μl. The samples were incubated at 30 °C overnight with shaking at 800 rpm and analyzed by HPLC as described above.

### Enzyme-catalyzed hydrogen/deuterium exchange assays

An excess of indole (2 mM) was mixed with 50 mM KPi pD 6.0 in D_2_O to obtain a saturated solution of indole in D_2_O. After the addition of AnInD to a final concentration of 2 μM, the ^1^H NMR spectra were recorded at 298 K on a Bruker 500 MHz AVIII NMR spectrometer with QCI-F cryoprobe equipped with z-gradients, using the noesygppr1d pulse sequence for water signal suppression (1.7s acquisition time, 2s interscan delay, 90^°^^1^H pulses). Dead time between enzyme addition and recording of spectra was approximately 20 min, except where indicated.

### EPR spectroscopy

EPR spectra were obtained using a Bruker ELEXSYS E500 EPR spectrometer equipped with a Super High Q (ER 4118-SHQ) resonator coupled to an Oxford Instruments ESR900 helium flow cryostat for temperature control. Spectra were acquired at 20 K using 10 μW microwave power, 100 KHz modulation frequency, and 1 G modulation amplitude.

### Substrate screening

A small library of heteroaromatic compounds (I3C, indole-2-carboxylate, pyrrole-2-carboxylate, indazole-3-carboxylate, quinoxaline-2-carboxylate, quinoline-2-carboxylate, indene-3-carboxylate, benzothiophene-3-carboxylate, benzothiophene-2-carboxylate, 2-naphthoic acid, and 1-naphthoic acid) was screened against AnInD (both WT and variants). The samples were incubated at 30 °C overnight with shaking at 800 rpm, and HPLC was used for the analysis with the relevant λ_max_ used for product detection. All the assays were performed as described above.

### One pot synthesis of indole-3-carboxyaldehyde by coupling AnInD and SrCAR

A one pot coupling reaction was performed in 50 mM potassium phosphate buffer final pH 7.0 and 8.0, containing 2 mM NADPH, 2 mM ATP, 2 mM MnCl_2_, 10 mM MgCl_2_, 5 mM indole, and various NaHCO_3_ concentrations (0.25, 0.5, 0.75 M, and 1 M) to determine the optimum reaction conditions. Reactions were carried out in a total volume of 500 μl in 2 ml crimp sealed amber vials. All the reactions were incubated at 30 °C with shaking at 800 rpm and quenched by the addition of an equal volume of acetonitrile containing 0.1% TFA. The samples were centrifuged at 16,100*g* to remove precipitated protein and the supernatant transferred to HPLC glass vials. Samples were analyzed using an Agilent 1260 Infinity Series HPLC equipped with a diode array UV detector as described above.

### Crystallization, data collection, and structure determination

AnInD was freshly purified and concentrated to *ca* 15 mg/ml before setting down for crystallization trials. Initial crystallization screening was performed with a mosquito nano-dispenser (TTP LabTech) in sitting drop-vapor diffusion plates using commercially available crystallization screens (PACT, JCSG, SG1, Morpheus I & II) (Molecular Dimensions). AnInD crystals appeared overnight in a number of conditions across the screens at 20 °C. The best diffracting crystals of AnInD appeared in the G5 condition of the PACT screen. Crystals were cryo-protected in reservoir solution supplemented with 10% PEG_200_ and flash frozen in liquid nitrogen. Diffraction data was collected at Diamond beamlines and processed using the CCP4 suites (33). The AnInD hexamer structure was solved by molecular replacement implemented in Phaser (34) using ShVdcCD (PDB id: 7AE5). Molecular replacement solution was determined with the core region of ShVdcC hexamer, composed of six oligomerization domains from the functional ShVdcC hexamer. All six prFMN-binding domains were found by searching ShVdcC prFMN-binding domain on the previously obtained partial molecular replacement solution. Automated model-building and refinement was carried out on the molecular replacement solution using Phenix.autobuild. Iterative cycles of manual model building in Coot followed by refinement using Phenix.refine were used to complete the AnInD structure (33, 35).

### Solution studies by SAXS

Small-angle x-ray scattering intensity data, I(q) *versus* q, were collected using HPLC SAXS on beamline B21 at Diamond Light Source. A Shodex KW-403 column mounted on an Agilent HPLC, with a 0.15 ml min^-1^ flow rate, coupled to a SAXS beam, was used for inline SAXS data collection. Small-angle x-ray scattering data was collected at one-second intervals using a Pilatus 2M detector (Dectris) at a distance of 3.9 m and an X-ray wavelength of 1 Å. All the SAXS data sets were analyzed by ATSAS (36) and Scatter suite (37). The FoXS web server was used to assess the fitting of SAXS data with the corresponding models (38, 39). Rigid body models of the open and closed states of the hexamer were produced by docking of the closed model of AnInD on crystal structure of AnInD. For each conformer, hydrodynamic parameters were calculated with SOMO (40).

## Data availability

The atomic coordinate and structure factor (pdb code: 7P9Q) have been deposited to the Protein Data Bank (http://www.pdb.org).

## Supporting information

This article contains supporting information.

## Conflict of interest

The authors declare that they have no conflict of interest with the contents of this article.
