# Supplementary material for: Structural and biochemical characterization of the prenylated flavin mononucleotide-dependent indole-3-carboxylic acid decarboxylase
Source: J Biol Chem. 2022 Feb 24;298(4):101771. doi: 10.1016/j.jbc.2022.101771 (PMC8988006; doi:10.1016/j.jbc.2022.101771)
Supplement: Supplementary Figures S1–S5 and Table 2 [file mmc1.docx]

**Supporting Information**

**Structural and biochemical characterisation of prFMN-dependent indole-3-carboxylic acid decarboxylase**

Deepankar Gahloth^1^, Karl Fisher^1^, Karl A.P. Payne^1^, Matthew Cliff^1^, Colin Levy^1^, David Leys^1*^

^1^ Manchester Institute of Biotechnology, University of Manchester, 131 Princess Street, Manchester, M1 7DN, UK.

**Supplementary Figure S1.** pH screening of AnInD and SrCAR coupling one-pot reaction

**Supplementary Figure S2**. Amino acid sequence alignment.

**Supplementary Figure S3.** Superposition of AnInD open crystal structure on AnInD closed model.

**Supplementary Figure S4**. SAXS analysis of shape and flexibility of AnInD.

**Supplementary Figure S5**. Cartoon representation of possible combination of open and closed conformations in AnInD hexamer.

**Supplementary Table S1**. Summary of kinetic parameters of AnInD purified in different conditions.

**Supplementary Figure S1**

**Supplementary Figure S1**. One pot synthesis of Indole 3-carboxyaldehyde by coupling of AnInD and SrCAR activity. Assays contain 5μM AnInD, 5mM indole, 4mM ATP, 3mM NADPH, and 2 μM SrCAR. NaHCO_3_ concentration was increased from 0.25 M to 1 M in 100mM Kpi pH 6.5 (〇; actual pH 0.25 M = 7, 0.5 M = 7.3, 0.75 = 7.4, 1 M = 7.6) and 100 mM Kpi pH 7.5 (∆; all NaHCO_3_ concentration = pH 8.0).

**Supplementary Figure S2**

**
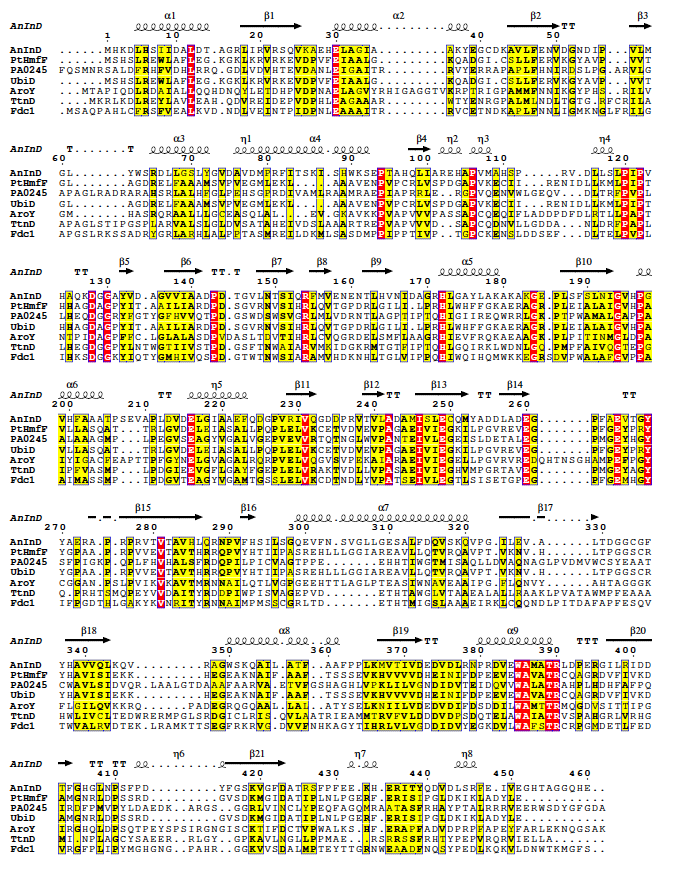
**

**Supplementary Figure S2**. Amino acid sequence alignment of AnInD, PtHmfF (PDB id: 6H6V), PA0245 (PDB id: 7ABO), E.coli UbiD (PDB id:5M1E ), AroY (PDB id: 5O3N), TtnD (PDB id: 6DA9) and Fdc1 (PDB id: 4ZAB). Conserved residues are highlighted in red. Secondary structure assignment was shown on top of the alignment for AnInD.

**Supplementary Figure S3**


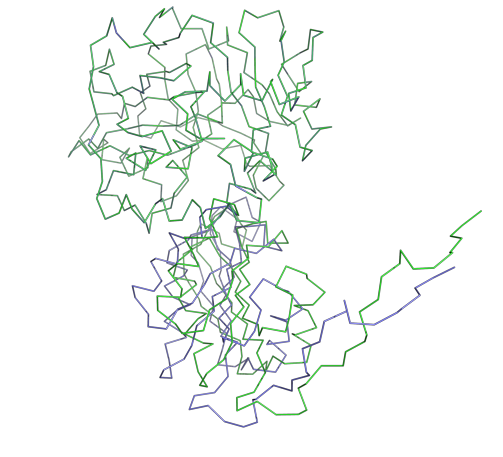


**Supplementary Figure S3.** Superposition of AnInD open crystal structure (monomer) on to the closed model. Closed AnInD model was modelled by rigid body rotation of dimerization domain on G310 hinge region. AnInD open crystal structure shown in blue and closed model shown as green ribbon.

**Supplementary Figure S4**

**Supplementary Figure S4. (A)** SAXS Analysis of shape and flexibility for AnInD. Guinier fitting plots show the logarithm of the X-ray intensity **ln [I(q)]** as a function of the square of the modulus of the scattering vector **q^2^** (in Å^-2^) for AnInD. The radii of gyration (*Rg*) measured from the slopes of the linear regions at low values of q^2^ are: 4.8 nm for AnInD. Experimental data are shown as red empty circles and the lines of best fit are show in red. Residual plots show random distributions of differences between experimental values and those calculated from the lines of best fit. **(B)** P(r) distribution plots show the probability of distance between scatterers **p(r)** against the distance **r** (in Å) for AnInD. The maximum inter-scatterer distances *Dmax* is 13.2 nm for AnInD. **(C)** Dimensionless Kratky plots show **I(q)/I(0) × (q×*Rg*)^2^** against **q×*Rg*** where **I(q)/I(0)** is the normalised X-ray scattering intensity, ***Rg*** is the radius of gyration in Å and **q** is the modulus of the scattering vector in Å^-1^. **(D)** The Porod-Debye plots show **q^4^ × I(q)** against **q^4^** whereas **(E)** SIBYLS plots show **q^3^ × I(q)** against **q^3^**, where **I(q)** is the X-ray intensity and **q** is the modulus of the scattering vector in Å^-1^. Data reaching a plateau at high q4 values in the Porod-Debye plot would indicate compact particles. The AnInD plots are consistent with compact particles in solution. Graphs are prepared using ScÅtter (http://www.bioisis.net/tutorial/9).

**Supplementary Figure S5**


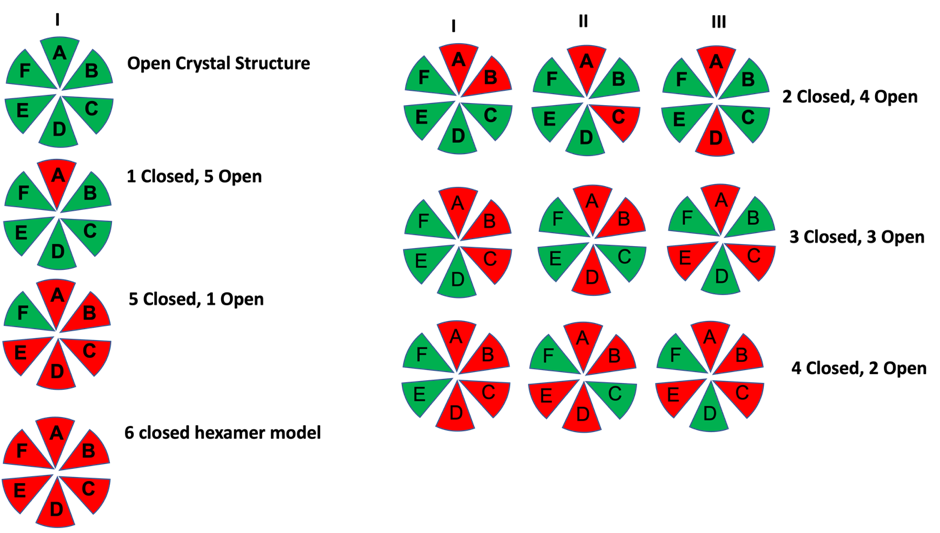


**Supplementary Figure S5**: Cartoon representation of possible combinations of open and closed conformation of monomers in AnInD hexamer in solution. AnInD hexamer is represent by six shapes and each monomer labelled from A to F (1-6). Green represents open conformation as observed in crystal structure, red represents closed conformation modelled by the rigid body rotation of oligomerization domain at hinge G310. Provided domain motion in the hexamer functional unit of AnInD, other intermediate conformation is also possible.

**Supplementary Table S1**.

Summary of the kinetics of AnInD as-purified, reconstituted and titrated with anaerobic and aerobic substrate (Indole 3-Carboxylic acid) in various conditions. Aerobic substrate solution was prepared on bench and used in both bench and glove box. Anaerobic substrate was dissolved in nitrogen purge DMSO in glove box to keep the substrate anaerobic. Aerobically purified protein was assessed with aerobic substrate only. All the data represented is done in triplicate and expressed as k_obs_(s^-1^).
